# Intracranial Pressure Variability and Low Cerebral Perfusion Pressure Burden as Prognostic Markers Beyond Mean Pressure in Neurocritically Ill Patients

**DOI:** 10.3390/jcm15187056

**Published:** 2026-09-11

**Authors:** Jihyuk Chung, Jeong-Am Ryu

**Affiliations:** 1 Department of Thoracic and Cardiovascular Surgery, Samsung Medical Center, Sungkyunkwan University School of Medicine, 81 Irwon-ro, Gangnam-gu, Seoul 06351, Republic of Korea; jihyuk.chung@samsung.com; 2 Department of Neurosurgery, Samsung Medical Center, Sungkyunkwan University School of Medicine, 81 Irwon-ro, Gangnam-gu, Seoul 06351, Republic of Korea; 3 Department of Critical Care Medicine, Samsung Medical Center, Sungkyunkwan University School of Medicine, 81 Irwon-ro, Gangnam-gu, Seoul 06351, Republic of Korea

**Keywords:** intracranial pressure, cerebrovascular circulation, critical care, prognosis, Glasgow outcome scale, treatment outcome

## Abstract

**Background/Objectives:** Elevated intracranial pressure (ICP) drives secondary brain injury and poor outcome. The pressure reactivity index (PRx), the reference marker of cerebral autoregulation, requires high-frequency waveforms unavailable in many centers. We examined whether routinely charted hourly ICP and cerebral perfusion pressure (CPP) can stratify prognosis without the PRx. **Methods:** In a retrospective cohort of 990 adults with invasive ICP monitoring at a tertiary neurosurgical ICU (a predominantly non-traumatic case mix), we derived from hourly ICP the mean, variability (within-patient standard deviation), and peak burden (time above 22 mmHg), and from CPP the low-perfusion burden (time below 60 mmHg). Each metric (per 1 SD) was related to 28-day mortality and poor neurological outcome (Glasgow Outcome Scale 1–3), adjusted for age, sex, APACHE II, GCS, diagnosis, and mean ICP, with incremental value and sensitivity analyses. **Results:** In total, 86 of 990 patients died within 28 days. ICP variability was associated with both mortality and poor neurological outcome and, unlike peak burden, remained independent after adjustment for mean ICP. Peak burden lost significance once mean and variability were known. For CPP, hypoperfusion burden, but not CPP variability, was associated with mortality. CPP variability did not survive joint modeling with ICP variability. Excess risk was confined to low CPP, with no harm at high CPP. ICP variability added incremental value beyond a clinical model, robust to sensitivity analyses. **Conclusions:** Routinely available ICP variability and CPP hypoperfusion burden carry prognostic information beyond mean pressure. ICP variability predicted both outcomes, whereas hypoperfusion burden predicted mortality. These summaries may offer a pragmatic alternative where the PRx is unavailable, pending validation.

## 1. Introduction

Elevated intracranial pressure (ICP) is a principal cause of secondary brain injury and is consistently associated with poor outcome across neurocritical illness [1]. Its control is therefore a central goal of neurocritical care, and contemporary guidelines organize management around the mean pressure and the exceedance of fixed thresholds [2].

The prognostic information carried by ICP may, however, extend beyond its average level. Beyond a sustained elevation, the temporal instability of pressure—its variability—has been linked to impaired cerebrovascular reactivity and to the slow-wave dynamics that reflect the brain’s compensatory reserve [3,4,5,6]. The conceptual reference standard for capturing this dimension is the pressure reactivity index (PRx), a continuous correlation between ICP and arterial pressure that indexes cerebral autoregulation [3].

The clinical reach of the PRx is nonetheless limited. Its computation requires high-frequency, time-synchronized ICP and arterial blood pressure waveforms together with dedicated software, which are unavailable in many intensive care units. As a result, autoregulation-based prognostication remains largely confined to specialized centers, while the majority of clinicians rely on the hourly ICP and cerebral perfusion pressure (CPP) values that are routinely charted [7,8].

Whether these routinely available summaries can themselves stratify prognosis is not established. Hourly ICP permits estimation not only of the mean but also of the variability in and the burden of pressure peaks, whereas CPP—the difference between mean arterial pressure and ICP—reflects the adequacy of cerebral perfusion. For CPP, it remains unresolved whether prognosis is better captured by its variability or by the burden of hypoperfusion. Both are plausible, since unstable perfusion and sustained perfusion below the ischemic threshold could each contribute to injury [9,10].

We therefore conducted a retrospective cohort study to determine whether routinely charted ICP variability and peak burden, together with CPP hypoperfusion burden, provide prognostic information beyond mean pressure, and whether they add incremental value to a clinical model. We framed these summaries as a pragmatic alternative to autoregulation-based indices in settings where high-frequency waveform monitoring is not feasible. Because recommendations for ICP monitoring derive almost entirely from traumatic brain injury, whereas much real-world monitoring occurs in non-traumatic conditions, we deliberately examined a predominantly non-traumatic neurosurgical cohort, interpreting thresholds and concepts imported from the trauma literature with this distinction in mind.

## 2. Materials and Methods

### 2.1. Study Design and Setting

This was a single-center, retrospective cohort study conducted at Samsung Medical Center, a tertiary academic hospital in Seoul, South Korea. We reviewed adult patients (aged ≥18 years) admitted to the neurosurgical intensive care unit (ICU) who underwent invasive intracranial pressure (ICP) monitoring between January 2016 and April 2026. The study was approved by the Institutional Review Board of Samsung Medical Center (IRB No. SMC 2026-07-047-001), and the requirement for informed consent was waived owing to the retrospective observational design.

Clinical data were retrospectively extracted from the hospital’s clinical data warehouse (CDW), “Darwin-C,” an institutional research platform that enables investigators to search and retrieve de-identified medical records from the electronic archives of more than 4 million patients. Demographic, clinical, laboratory, physiologic time series, and outcome data were extracted from the CDW after the study cohort was finalized.

### 2.2. Study Population and Definition

We identified all ICU admissions during the study period in whom invasive ICP monitoring was performed. Of 992 patients screened, 990 were included in the analytic cohort; 2 patients were excluded because of absent or uninterpretable hourly intracranial pressure recordings that precluded computation of ICP metrics (Supplementary Figure S1).

The decision to initiate invasive ICP monitoring was made by the attending neurosurgeons and neurointensivists rather than by a fixed protocol. Typical indications were (i) acute hydrocephalus or intraventricular hemorrhage requiring therapeutic cerebrospinal fluid diversion—for example, after subarachnoid or intracerebral hemorrhage or in patients with brain tumors—in which the external ventricular drain served both treatment and monitoring [11,12,13]; (ii) planned postoperative monitoring after major intracranial surgery, particularly extensive tumor resection with anticipated brain edema; and (iii) depressed consciousness or radiographic mass effect limiting serial neurological examination, with the small number of patients with traumatic brain injury managed according to existing guidelines. When cerebrospinal fluid diversion was not required, intraparenchymal or epidural sensors were used.

The admission Glasgow Coma Scale (GCS) was defined as the best (highest) total score documented within the first 12 h of ICU admission, a definition chosen to minimize the influence of transient sedation and anesthesia. The total score was recalculated from the individual eye, verbal, and motor subscores extracted from the detailed neurological assessment records. For patients in whom the verbal component was unassessable owing to endotracheal intubation or tracheostomy, the verbal score was imputed using the Rutledge regression method [14], which predicts the verbal score from the eye and motor subscores. Patients lacking the individual subscores required for this correction were excluded from GCS-based analyses. The verbal score imputation changed the total score in 48 patients (5.3% of those with available subscores), and the GCS was missing in 90 patients (9.1%).

ICP was monitored invasively using an external ventricular drain, an intraparenchymal fiberoptic monitor (Camino; Integra LifeSciences, Plainsboro, NJ, USA), an epidural monitor (Spiegelberg GmbH & Co. KG, Hamburg, Germany), or, in a small minority of patients, a lumbar drain, in accordance with contemporary neurocritical care practice [2]. Lumbar drains (12 patients, 1.2%) were placed at the discretion of the treating neurosurgeons in patients judged to have communicating cerebrospinal fluid pathways, typically for therapeutic cerebrospinal fluid drainage, and the lumbar pressure was charted as the ICP in these patients. Because lumbar cerebrospinal fluid pressure approximates intracranial pressure only under communicating conditions and is not interchangeable with it in the presence of mass lesions or obstructed cerebrospinal fluid pathways, a sensitivity analysis excluding these patients was performed (Section 3.6). Hourly ICP values were not captured automatically from the bedside monitor: at each hour, the displayed value—after leveling of the transducer at the external auditory meatus—was read and entered manually into the electronic nursing record by trained ICU nurses, so that each hourly value represents a single-point measurement rather than a down-sampled waveform. Hourly ICP charted in this unit has been analyzed previously [15,16]. In patients with an external ventricular drain, the catheter served both cerebrospinal fluid diversion and pressure monitoring, and no standardized clamping protocol was applied before readings [11,17]. Non-numeric entries and values outside the physiologic range (−10 to 100 mmHg) were excluded (0.34% of charted values). Hourly ICP values recorded during the ICU stay were then extracted. From each patient’s ICP time series, we derived three complementary dimensions. The level was summarized by the mean ICP. Variability was quantified as the within-patient standard deviation (SD) of hourly ICP; complementary variability metrics (coefficient of variation and the root mean square of successive differences) were computed for sensitivity analyses. Burden was quantified as the proportion of monitored time with ICP above 22 mmHg and as the cumulative pressure–time dose (the integral of ICP above threshold). To evaluate early-phase prognostication and to mitigate immortal time bias, metrics were computed over the entire monitoring period and, separately, within the first 24 and 72 h. All time-based metrics were normalized to the monitored duration. Full definitions of all ICP and CPP metrics are provided in Supplementary Table S1.

For patients with concurrently available blood pressure data, CPP was calculated as the difference between mean arterial pressure (MAP) and ICP. MAP was derived preferentially from invasive arterial catheter recordings. When arterial recordings were unavailable, non-invasive measurements were used, with MAP calculated as the diastolic pressure plus one-third of the pulse pressure. Each MAP value was matched to the nearest hourly ICP measurement within ±30 min. CPP low-perfusion burden was quantified as the proportion of time with CPP below 60 mmHg [2], and CPP variability was quantified analogously to ICP. A CPP above 60 mmHg was also the explicit institutional management target throughout the study period [15]. Because arterial pressure was charted more sparsely than ICP—predominantly during periods of arterial catheterization—CPP metrics were derived on an available-case basis, and the potential for informative sampling was addressed in sensitivity analyses using inverse probability weighting. Additional analyses examined alternative ICP peak burden thresholds (20, 25, and 30 mmHg); adjustment for the primary monitoring device; adjustment for major treatment exposures (hypertonic saline, mechanical ventilation, renal replacement therapy, neurosurgical operation, and decompressive craniectomy); models omitting the admission GCS; multiply imputed incremental models; and poor neurological outcome restricted to patients alive at hospital discharge. The primary hypothesis was the association of ICP variability with 28-day mortality. For the twelve pre-specified adjusted exposure–outcome association tests (six pressure metrics against the two outcomes), *p* values were additionally corrected for multiplicity using the Holm procedure, which controls the family-wise error rate, and the Benjamini–Hochberg false-discovery-rate procedure (Supplementary Table S12). All remaining comparisons were exploratory and are interpreted accordingly.

The primary outcome was 28-day all-cause mortality from ICU admission. Secondary outcomes were poor neurological outcome, defined as a Glasgow Outcome Scale (GOS) score of 1–3 (death, persistent vegetative state, or severe disability) [18], in-hospital mortality (death before hospital discharge), ICU mortality, and all-cause mortality at any time during the available follow-up (any death recorded in the clinical data warehouse, including after discharge). The GOS was classified from the neurological status documented at hospital discharge—an early, non-uniform time point—rather than at a fixed follow-up interval.

### 2.3. Data Collection

Baseline characteristics collected at ICU admission included demographics (age, sex), ICU admission diagnosis, severity of illness (admission GCS and Acute Physiology and Chronic Health Evaluation [APACHE] II score [19]), comorbidities, and ICU management variables (mechanical ventilation, continuous renal replacement therapy, and ICP monitoring). Admission diagnosis was grouped into four categories (brain tumor, intracerebral hemorrhage, subarachnoid hemorrhage, and other) for adjustment.

### 2.4. Statistical Analysis

Continuous variables are presented as median (interquartile range) and compared using the Mann–Whitney U test. Categorical variables are presented as *n* (%) and compared using the chi-square or Fisher’s exact test, as appropriate. Standardized mean differences were used to compare distributions across outcome groups.

To evaluate the independent prognostic association of each ICP and CPP metric, multivariable logistic regression models were fitted with each metric entered per 1 SD increment. The adjustment set comprised age, sex, APACHE II score, admission GCS, and diagnostic category, following the principle of at least 10 events per variable to limit overfitting [20]. Because pressure variability is correlated with the mean, mean ICP was additionally included so that the independent contribution of variability could be assessed after accounting for the level. The incremental predictive value of variability and burden beyond a clinical model and mean pressure was evaluated using hierarchical models constructed by sequentially adding the mean ICP, ICP variability, peak burden, and CPP low-perfusion burden. At each step, the added metric was evaluated relative to the immediately preceding model. Model performance was compared using the change in the C-statistic [21], the likelihood ratio test (the primary criterion of incremental fit), the integrated discrimination improvement (IDI), and the continuous net reclassification improvement (NRI) [22], with 95% confidence intervals obtained by bootstrap resampling. Confidence intervals for the NRI were obtained from 1000 bootstrap resamples. Full definitions and computational details are provided in the Supplementary Methods. Dose–response relationships between continuous metrics and outcome risk were examined using restricted cubic splines (natural cubic basis, three degrees of freedom, knots at quantile-based positions) [23].

Several sensitivity analyses were performed. To address immortal time bias, associations were re-estimated using metrics restricted to the first 72 h [24]. The robustness of significant associations to unmeasured confounding was quantified using the E-value [25]. To address the potential selection bias arising from the availability of arterial pressure data, CPP analyses were repeated with inverse probability weighting [26]. Missing values in APACHE II score and admission GCS were addressed in sensitivity analyses using multiple imputation by chained equations [27], and rare-event models used Firth’s penalized likelihood [28].

We did not attempt to measure cerebral autoregulation or brain compliance. The analysis was confined to routinely available ICP and CPP summaries. All statistical tests were two-sided, and *p* < 0.05 was considered statistically significant. Analyses were performed using Python 3.10.12 with statsmodels 0.14.6, lifelines 0.30.0, and scikit-learn 1.7.2.

## 3. Results

### 3.1. Study Population

Of 14,572 ICU patients screened during the study period, 992 underwent invasive ICP monitoring. After exclusion of two patients with absent or uninterpretable ICP recordings, 990 patients formed the analytic cohort (Supplementary Figure S1). CPP was derived in 950 patients (96.0%), GOS was available in 917 patients, of whom 218 (23.8%) had a poor neurological outcome (GOS 1–3), and 856 patients had complete data for the incremental models. Within 28 days, 86 of 990 patients (8.7%) died. Ninety-six patients (9.7%) died before hospital discharge (in-hospital mortality) and 57 (5.8%) died in the ICU. In total, 271 patients (27.4%) died at any time during the available follow-up, including deaths a median of approximately one year after discharge among patients discharged in good neurological condition. The distribution of the discharge GOS was 96 (GOS 1), 10 (GOS 2), 112 (GOS 3), 173 (GOS 4), and 526 (GOS 5), with 73 not classifiable. GOS 1 corresponded exactly to in-hospital death, and 122 survivors had GOS 2–3 (Supplementary Table S7). ICP was monitored via an external ventricular drain in 857 patients (86.6%), an epidural sensor in 115 (11.6%), a lumbar drain in 12 (1.2%), and an intraparenchymal sensor in six (0.6%). Hypertonic saline was administered in 869 patients (87.8%), mechanical ventilation in 51.4%, renal replacement therapy in 1.5%, and a neurosurgical operation was performed during the index admission in 758 patients (76.6%), including decompressive craniectomy in 101 (10.2%).

### 3.2. Baseline Characteristics

Baseline characteristics according to 28-day survival are shown in Table 1. Compared with survivors, non-survivors were older (median 62 vs. 53 years, *p* < 0.001) and had higher APACHE II scores (28 vs. 23, *p* < 0.001), a higher rate of mechanical ventilation (93.0% vs. 47.5%, *p* < 0.001), and a different diagnostic distribution (intracerebral hemorrhage 36.0% vs. 5.9%; brain tumor 26.7% vs. 51.1%; *p* < 0.001). Non-survivors had higher mean ICP (14.2 vs. 11.4 mmHg), higher ICP variability (5.5 vs. 4.2 mmHg), a greater proportion of time with ICP > 22 mmHg (4.8% vs. 0.0%), lower mean CPP (72.8 vs. 78.5 mmHg), and a greater proportion of time with CPP < 60 mmHg (13.8% vs. 2.7%) (all *p* ≤ 0.004).

### 3.3. Intracranial Pressure Level, Variability, and Peak Burden

**Figure 1:**
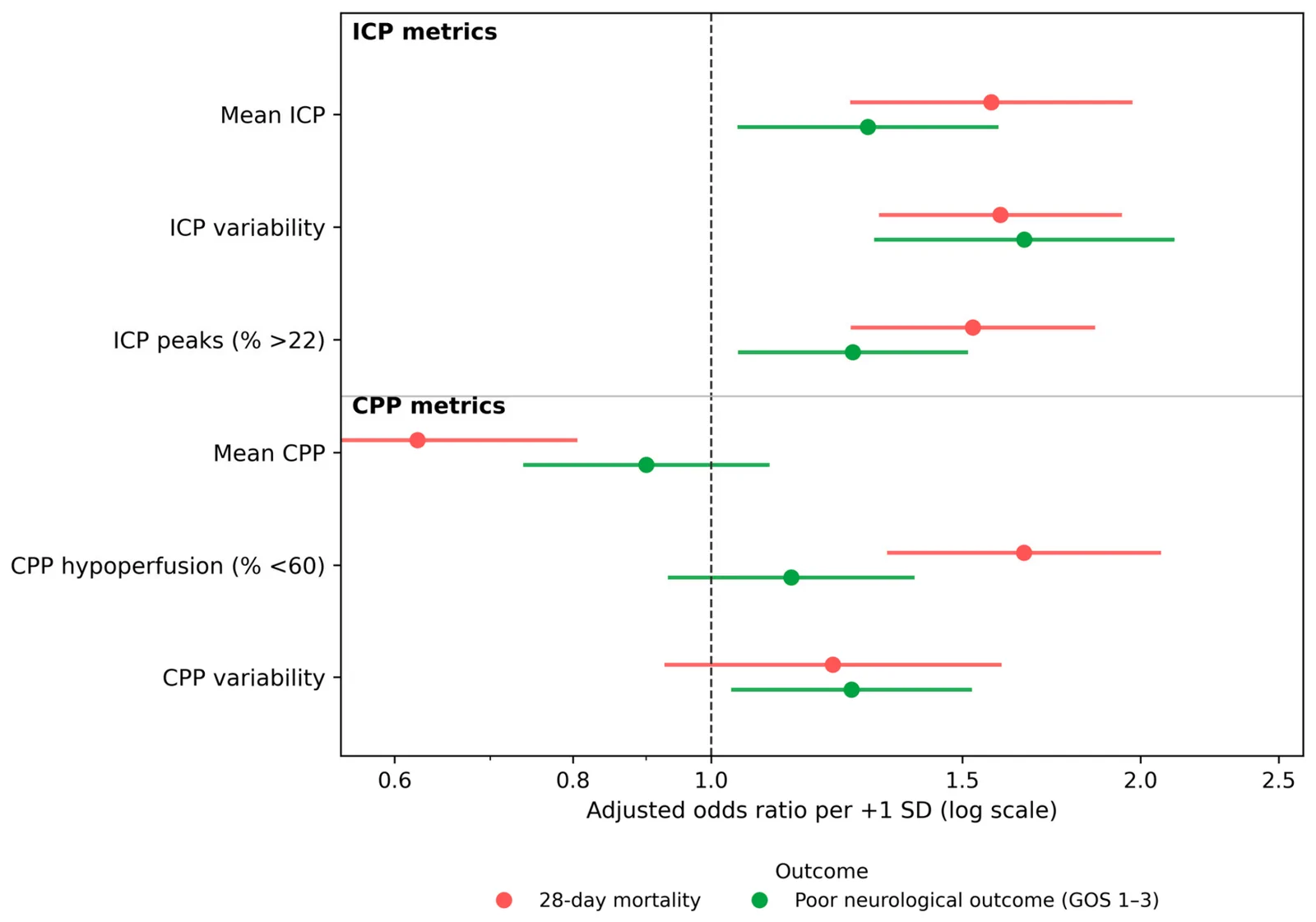
Adjusted associations of ICP and CPP summary metrics with 28-day mortality (red) and poor neurological outcome, GOS 1–3 (green). Each marker is the odds ratio per +1 SD increment from a multivariable logistic model adjusted for age, sex, APACHE II, admission GCS (corrected), and diagnosis category; horizontal bars are 95% CIs. “Variability” denotes the within-patient SD of ICP/CPP. ICP variability was associated with both outcomes, whereas CPP variability was not; CPP contributed mainly through low mean CPP and hypoperfusion burden. Abbreviations: APACHE II, Acute Physiology and Chronic Health Evaluation II; CI, confidence interval; CPP, cerebral perfusion pressure; GCS, Glasgow Coma Scale; GOS, Glasgow Outcome Scale; ICP, intracranial pressure; SD, standard deviation.

Associations of each ICP metric with outcomes, adjusted for age, sex, APACHE II score, admission GCS, and diagnosis, are shown in Table 2 and Figure 1. Per 1 SD increment, mean ICP was associated with 28-day mortality (adjusted odds ratio [aOR] 1.57, 95% CI 1.26–1.97, *p* < 0.001) and with poor neurological outcome (aOR 1.29, 95% CI 1.05–1.58). ICP variability was associated with 28-day mortality (aOR 1.59, 95% CI 1.32–1.93, *p* < 0.001) and with poor neurological outcome (aOR 1.66, 95% CI 1.30–2.11), and its association with 28-day mortality persisted after additional adjustment for mean ICP (aOR 1.48, 95% CI 1.19–1.84, *p* < 0.001). ICP peak burden (percentage of time > 22 mmHg) was associated with 28-day mortality (aOR 1.53, 95% CI 1.26–1.85, *p* < 0.001; Table 2) but added no incremental value once mean ICP and variability were included (Table 3). The coefficient of variation of ICP was not associated with 28-day mortality (aOR 1.00, *p* = 0.99; Supplementary Figure S2; Supplementary Table S3). Associations of peak burden were consistent across alternative thresholds of 20, 25, and 30 mmHg (Supplementary Table S9).

### 3.4. Cerebral Perfusion Pressure

**Figure 2:**
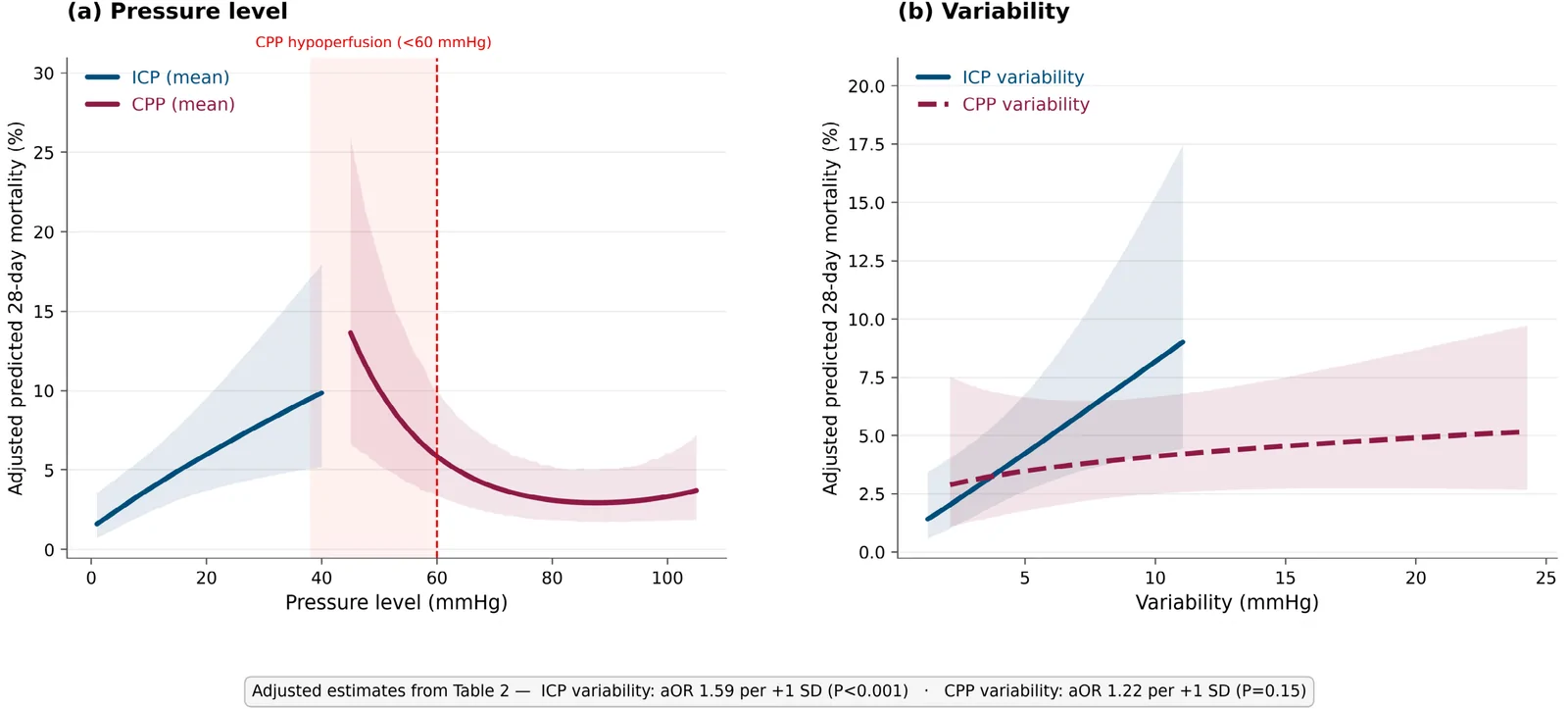
Adjusted dose–response of pressure level (**a**) and variability (**b**) with 28-day mortality. (**a**) Predicted mortality rose with mean ICP and fell with increasing mean CPP, with excess risk confined to hypoperfusion (<60 mmHg, shaded) and no harm at high CPP. (**b**) Predicted mortality increased significantly with ICP variability (aOR 1.59 per +1 SD, *p* < 0.001) but not with CPP variability (aOR 1.22, *p* = 0.15). Curves adjusted for age, sex, APACHE II, admission GCS (corrected), and diagnosis; each variability curve additionally adjusted for its own mean. “Variability” = within-patient SD; bands are 95% CIs. Abbreviations: APACHE II, Acute Physiology and Chronic Health Evaluation II; CI, confidence interval; CPP, cerebral perfusion pressure; GCS, Glasgow Coma Scale; ICP, intracranial pressure; OR, odds ratio; SD, standard deviation.

CPP metrics are shown in Table 2. Per 1 SD increment, higher mean CPP was associated with lower 28-day mortality (aOR 0.62, 95% CI 0.48–0.80, *p* < 0.001). Low-perfusion burden (percentage of time < 60 mmHg) was associated with 28-day mortality (aOR 1.66, 95% CI 1.33–2.06, *p* < 0.001) but not with poor neurological outcome (aOR 1.14, 95% CI 0.94–1.38). CPP variability was not associated with 28-day mortality (aOR 1.22, 95% CI 0.93–1.59, *p* = 0.15). In a model including both ICP and CPP variability, ICP variability remained associated with 28-day mortality (aOR 1.46, 95% CI 1.20–1.78, *p* < 0.001), whereas CPP variability did not (aOR 1.10, 95% CI 0.84–1.45, *p* = 0.48). In a dose–response analysis with CPP 70–80 mmHg as the reference, only CPP < 60 mmHg was associated with increased 28-day mortality (aOR 6.62, 95% CI 2.51–17.42, *p* < 0.001); CPP 60–70 (aOR 1.85, *p* = 0.16), 80–90 (aOR 1.12, *p* = 0.79), and ≥90 mmHg (aOR 1.37, 95% CI 0.59–3.19, *p* = 0.47) were not (Supplementary Table S2). The adjusted dose–response relationships of pressure level and variability with 28-day mortality are shown in Figure 2. After correction for multiplicity across the twelve primary association tests, the five mortality associations with unadjusted *p* < 0.001 and the association of ICP variability with poor neurological outcome remained significant under the Holm procedure (all Holm-adjusted *p* ≤ 0.002), whereas the weaker associations of mean ICP, peak burden, and CPP variability with poor neurological outcome retained significance under the false-discovery-rate criterion (adjusted *p* = 0.024–0.027) but not under family-wise correction (Supplementary Table S12).

### 3.5. Incremental Predictive Value

**Figure 3:**
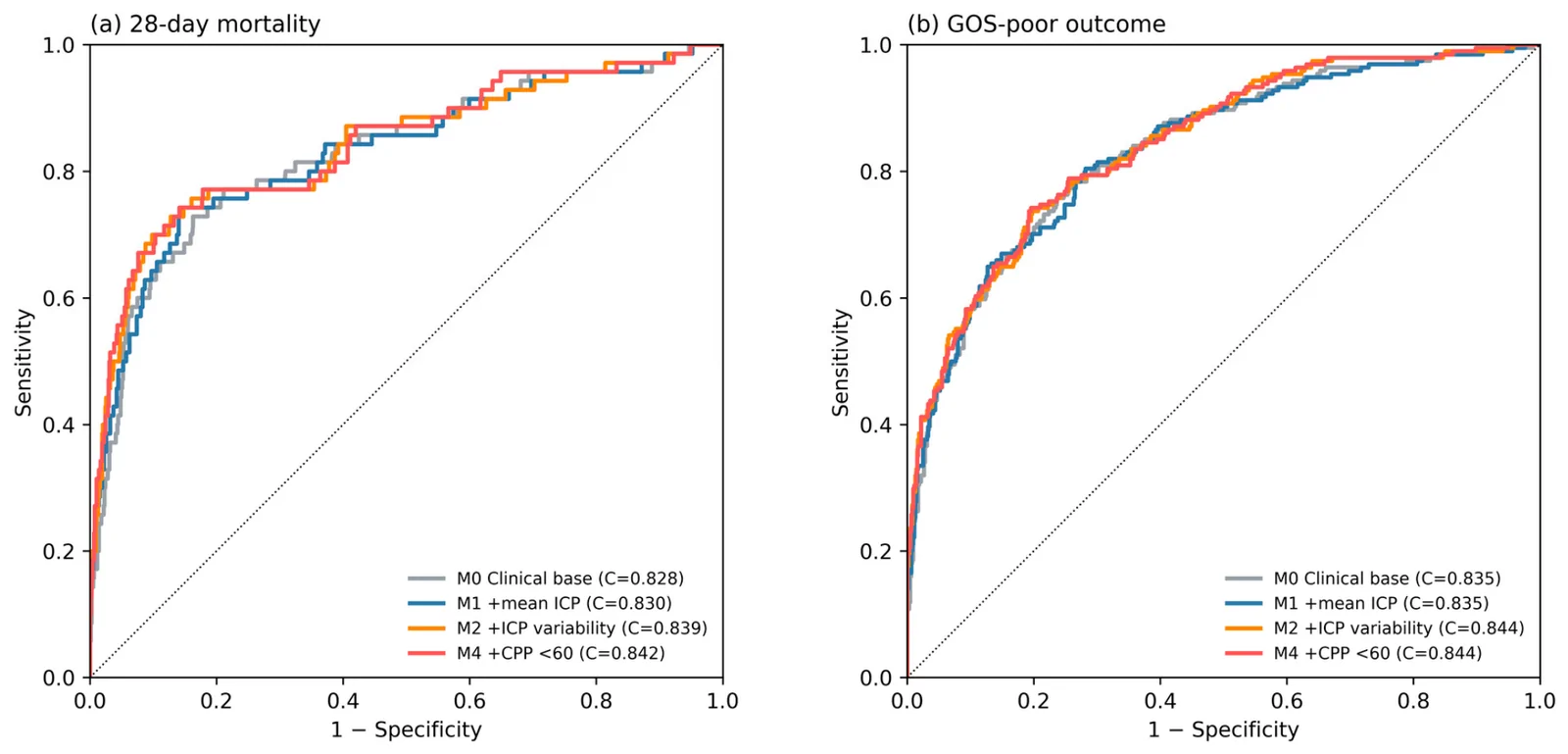
Incremental discrimination (ROC curves) of nested models for 28-day mortality (**a**) and poor neurological outcome (**b**). Sequentially adding ICP variability to a clinical base model plus mean ICP improved discrimination (see Table 3). C-statistics: M0, clinical base; M1, +mean ICP; M2, +ICP variability; M4, +CPP hypoperfusion (% time <60 mmHg). Abbreviations: CPP, cerebral perfusion pressure; ICP, intracranial pressure; ROC, receiver operating characteristic.

The incremental value of ICP and CPP metrics above a clinical model (age, sex, APACHE II score, admission GCS, diagnosis, and mean ICP) is shown in Table 3 and Figure 3. The clinical base model had a C-statistic of 0.828 (95% CI 0.773–0.885) for 28-day mortality and 0.835 (0.807–0.873) for poor neurological outcome. Adding ICP variability improved model fit for both 28-day mortality (C-statistic 0.839; likelihood ratio χ^2^ = 11.6, *p* < 0.001; IDI 0.026, *p* = 0.063) and poor neurological outcome (C-statistic 0.844; likelihood ratio χ^2^ = 20.1, *p* < 0.001; IDI 0.022, *p* = 0.009). Adding ICP peak burden did not improve fit (28-day mortality *p* = 0.56; poor neurological outcome *p* = 0.43). Adding CPP low-perfusion burden improved fit for 28-day mortality (*p* = 0.04) but not for poor neurological outcome (*p* = 0.57). These incremental fit conclusions rest primarily on the likelihood ratio test. In bootstrap analyses, the continuous NRI for adding ICP variability to the mortality model was +0.51 (95% CI +0.06 to +0.74), whereas the confidence intervals of all other reclassification estimates included zero (Table 3).

### 3.6. Sensitivity Analyses

Several sensitivity analyses were performed. First, the choice of variability metric was examined (Supplementary Figure S2; Supplementary Table S3). The within-patient standard deviation of ICP was used as the primary measure of variability, because the coefficient of variation—which normalizes to the mean—behaved inconsistently: the coefficient of variation of ICP was not associated with 28-day mortality (aOR 1.00, *p* = 0.99), whereas that of CPP was strongly associated (aOR 2.34, *p* = 0.005), the latter reflecting the correlation between a low mean CPP and a high coefficient of variation. The root mean square of successive differences in ICP was concordant with the standard deviation (aOR 1.54, *p* = 0.002). Second, to address immortal time bias, all metrics were recomputed using only the first 72 h of monitoring (Supplementary Figure S3). The association of ICP variability with 28-day mortality was essentially unchanged (aOR 1.60 over the entire monitoring period vs. 1.57 within the first 72 h), indicating that the association was not an artifact of longer observation among survivors. Third, the association of ICP variability with 28-day mortality was consistent across the major diagnostic categories, with no evidence of effect modification (brain tumor aOR 1.48 vs. non-tumor 1.77; interaction *p* = 0.97; Supplementary Figure S4). Fourth, robustness to unmeasured confounding was quantified with E-values (Supplementary Table S4). An unmeasured confounder would need to be associated with both the exposure and 28-day mortality by a risk ratio of at least 2.40 for ICP variability and 2.51 for CPP low-perfusion burden, beyond all measured covariates, to fully explain away the observed associations. The corresponding lower confidence limits were 1.88 and 1.91. Fifth, because CPP could be derived only in patients with concurrent arterial pressure recordings, the potential for selection bias was assessed (Supplementary Table S5). Patients with available CPP differed from those without, most notably in the duration of monitoring. Nonetheless, the association of CPP low-perfusion burden with 28-day mortality was unchanged after inverse-probability-of-selection weighting (unweighted aOR 1.60 vs. weighted 1.61). Sixth, the associations were compared across candidate outcomes (Supplementary Table S6). The associations of the acute pressure metrics were attenuated when all-cause mortality during the available follow-up (*n* = 271) was used instead of 28-day mortality—most markedly for ICP peak burden (aOR 1.53 vs. 1.08) and CPP low-perfusion burden (1.66 vs. 1.16)—whereas ICP variability remained associated with both endpoints (1.59 vs. 1.35). Seventh, associations of ICP variability with both outcomes were essentially unchanged after additional adjustment for the primary monitoring device (aOR 1.58 and 1.65) and for treatment exposures (aOR 1.55 and 1.62), as was CPP low-perfusion burden for 28-day mortality (aOR 1.71; Supplementary Table S10). Results were likewise unchanged when the admission GCS was omitted from the adjustment set (aOR 1.65 and 1.72). Excluding the 12 patients monitored via lumbar drain, in whom lumbar pressure only approximates intracranial pressure, likewise left the associations essentially unchanged (*n* = 978; ICP variability aOR 1.59 and 1.66; peak burden 1.51 and 1.26; CPP low-perfusion burden 1.67 for 28-day mortality; Supplementary Table S10). Eighth, in multiply imputed data, the added contribution of ICP variability was materially unchanged (pooled aOR 1.39 for 28-day mortality and 1.70 for poor neurological outcome), whereas the marginal contribution of CPP low-perfusion burden was attenuated (pooled *p* = 0.115; Supplementary Table S11). Finally, among patients alive at hospital discharge, ICP variability showed a directionally consistent, borderline association with poor neurological outcome (aOR 1.26, 95% CI 1.00–1.58, *p* = 0.050), whereas mean ICP and peak burden did not. In the same survivor-restricted model, CPP low-perfusion burden was inversely associated with poor neurological outcome (aOR 0.71, 95% CI 0.50–0.99, *p* = 0.042), a reversal of the main analysis that we interpret not as a protective effect but as index event (collider stratification) bias: restriction to survivor conditions on a variable strongly affected by the exposure, selecting against patients in whom a high hypoperfusion burden proved fatal and thereby inducing a spurious inverse association among those who remain (Supplementary Table S8).

## 4. Discussion

In this retrospective cohort of neurocritically ill patients monitored with ICP, two findings emerged. First, the variability in ICP carried prognostic information that was independent of, and incremental to, both the mean pressure and illness severity. Second, for CPP, the prognostically important feature was the burden of hypoperfusion rather than variability—most clearly for mortality—and high CPP was not associated with harm. Together, these define two complementary axes—pressure instability and hypoperfusion—that can be read from routinely charted data without high-frequency waveform analysis.

The independent association of ICP variability with outcome is biologically coherent. A brain with exhausted compensatory reserve transmits volume changes into larger pressure swings, so that variability reflects reduced intracranial compliance and the slow-wave activity that accompanies impaired cerebrovascular reactivity [3,4,5]. In this sense, hourly ICP variability may act as a low-resolution surrogate for the instability that the PRx captures at high resolution [6,7], while remaining computable from data that every monitored patient already generates. We deliberately refrain from equating variability with autoregulatory failure, because our data cannot measure reactivity directly. Variability is offered as a pragmatic prognostic marker rather than a mechanism.

The behavior of CPP was different and, at first glance, counterintuitive: although CPP integrates both arterial pressure and ICP, its variability added little once ICP variability was taken into account. Three considerations reconcile this. Physiologically, CPP variability is a composite of a brain-specific component (ICP) and a systemic component (arterial pressure). Much of the latter reflects hemodynamic manipulation and vasopressor titration rather than cerebral injury [29], so that the prognostic signal within CPP variability is largely inherited from its ICP component and is absorbed when ICP variability is modeled jointly. A complementary, more parsimonious account is measurement-related: arterial pressure was drawn from a mixture of invasive and non-invasive sources and matched to ICP within ±30 min, and this heterogeneity adds non-differential noise that would attenuate a CPP variability signal. Because ICP variability, measured at the same hourly resolution, retained a strong association, measurement noise alone is unlikely to explain the contrast, and the physiological and measurement accounts are best regarded as complementary. Clinically, what threatens the injured brain is not how much perfusion fluctuates but how often it falls below the ischemic threshold. Fluctuations above that threshold are generally tolerated, whereas sustained hypoperfusion drives injury [9,10]. Accordingly, the excess risk associated with CPP was confined to the hypoperfusion range, while higher CPP conferred no harm. This framing—perfusion adequacy rather than perfusion stability—positions low CPP burden, not CPP variability, as the second prognostic axis.

That these routine summaries added information on top of an already strong clinical model deserves comment, because when baseline discrimination is high, gains in global discriminative ability are inherently small and the value of a new marker is better judged by measures of model fit and reclassification [22,30]. By these measures, ICP variability improved the models for both outcomes, whereas the burden of pressure peaks did not once the mean and the variability were known. These observations are consistent with the possibility that, beyond the average level, the instability of pressure rather than the height of transient peaks carries independent prognostic information, although this inference from a retrospective analysis should be regarded as tentative and requires prospective confirmation.

Two further features of the cohort frame the interpretation. First, the case mix—brain tumors in roughly half, prior malignancy in almost three-quarters, and traumatic brain injury in fewer than 1%—reflects the referral pattern of a comprehensive cancer center and differs deliberately from the trauma cohorts in which ICP thresholds and autoregulation indices were developed. ICP data from predominantly non-traumatic populations are scarce, and monitoring in such patients—including those with brain tumors, in whom ventricular catheters have been used for pressure measurement since the earliest ICP studies—is supported by consensus recommendations rather than disease-specific trials [11]. Within this cohort, the imported thresholds behaved coherently: the excess risk of CPP was confined to values below 60 mmHg, which was also the institutional management target [15]; peak burden associations were consistent across alternative cutoffs; and the association of ICP variability was homogeneous across diagnostic categories. Second, the mortality structure matters: only 96 of the 271 deaths recorded during follow-up occurred before hospital discharge, and late deaths—occurring predominantly in patients discharged in good neurological condition and attributable mainly to the underlying disease—were poorly predicted by acute pressure metrics, supporting 28-day mortality as the primary outcome. Our findings extend observations from high-resolution traumatic brain injury cohorts, in which slow-wave activity and compensatory-reserve-weighted indices of ICP predict outcome [3,5,6,7], to hourly charted data in a predominantly non-traumatic population, and they complement the broader search for pragmatic approaches to intracranial pressure assessment where high-frequency or invasive monitoring is not available [31].

These observations may have a practical implication. Where high-frequency waveform monitoring and autoregulation indices are unavailable—which describes many intensive care units—the hourly ICP and CPP values already in the record might still carry prognostically meaningful information about both pressure instability and hypoperfusion. Such summaries are not a substitute for autoregulation-based management where it is feasible [7,8]. At most, they might be explored as a resource-light adjunct for risk stratification, a possibility that would need to be tested prospectively.

### Limitations

This study has several limitations. Its retrospective, single-center design and the predominance of patients with brain tumors limit generalizability, and unmeasured confounding cannot be excluded despite multivariable adjustment and quantitative bias analysis [25]. Hourly ICP values were manually charted single-point measurements: although non-physiologic values were rare (0.34%), reading and transcription errors cannot be excluded, and in patients with an external ventricular drain—86.6% of the cohort—the catheter served both drainage and monitoring without a standardized clamping protocol, so that values recorded during open drainage may not fully reflect intraparenchymal pressure [11,17]. Such error is expected to be largely non-differential and to bias variability estimates toward the null. Because the PRx was not measured concurrently, this study cannot determine how much of the information contained in high-frequency indices survives down-sampling to hourly values, and a prospective head-to-head comparison of hourly summaries against the PRx is needed. The admission GCS, although defined as the best value within 12 h, was usually obtained in sedated, ventilated patients (median 3) and may partly reflect sedation depth. The principal results were unchanged when the GCS was omitted from adjustment. Treatment intensity was only partially captured: osmotherapy, ventilation, renal replacement, and neurosurgical procedures were accounted for in sensitivity analyses, but sedation depth, cerebrospinal fluid drainage volume, hyperventilation, and treatment limitation decisions were not available, and treatment may act both as a confounder and as a mediator of the observed associations. Neurological outcome was classified from the neurological status at discharge rather than by prospective structured assessment and should be interpreted accordingly. Because this discharge-based classification was assessed at a non-uniform, early time point that may not reflect the eventual neurological trajectory, non-differential outcome misclassification could have attenuated associations with non-fatal neurological outcome—most relevant to CPP low-perfusion burden, whose prognostic signal was concentrated in mortality—whereas ICP variability remained associated with both outcomes. Structured long-term outcomes at a fixed interval (e.g., six months) could not be reconstructed from the records. The twelve primary association tests were corrected for multiplicity (Supplementary Table S12), whereas the wider set of exploratory and sensitivity comparisons was not, and borderline findings should be interpreted accordingly. CPP could be derived only in patients with concurrent arterial pressure monitoring, introducing the potential for selection, although weighting analyses did not materially change the findings. Finally, because monitoring began after admission, immortal time bias [24] may have influenced the estimates, and analyses restricted to the early monitoring window were used to mitigate this.

## 5. Conclusions

In conclusion, routinely available ICP variability and CPP hypoperfusion burden carried prognostic information beyond mean pressure in neurocritically ill patients—here, predominantly with non-traumatic diagnoses—capturing pressure instability and hypoperfusion as two complementary axes without requiring high-frequency waveform analysis. ICP variability was associated with both mortality and poor neurological outcome, whereas the prognostic value of CPP hypoperfusion burden was most evident for mortality. These findings are hypothesis-generating and warrant prospective, multicenter validation with structured long-term outcome measures before they inform clinical practice.

## Figures and Tables

**Table 1:** Baseline characteristics, by 28-day survival.

Characteristic	Overall (*n* = 990)	Survivors (*n* = 904)	Non-Survivors (*n* = 86)	SMD	*p*
Demographics					
Age, years	54.0 [39.0–64.0]	53.0 [38.0–63.0]	62.0 [54.2–73.0]	−0.68	<0.001
Male sex, *n* (%)	490 (49.5)	448 (49.6)	42 (48.8)	0.01	0.988
Primary diagnosis, *n* (%)				1.18	<0.001
Brain tumor	485 (49.0)	462 (51.1)	23 (26.7)	0.52	
Intracerebral hemorrhage	84 (8.5)	53 (5.9)	31 (36.0)	−0.80	
Subarachnoid hemorrhage	53 (5.4)	36 (4.0)	17 (19.8)	−0.50	
Ischemic stroke	12 (1.2)	10 (1.1)	2 (2.3)	−0.09	
Traumatic brain injury	8 (0.8)	7 (0.8)	1 (1.2)	−0.04	
CNS infection	12 (1.2)	11 (1.2)	1 (1.2)	0.00	
Other	336 (33.9)	325 (36.0)	11 (12.8)	0.56	
Comorbidities, *n* (%)					
Hypertension	117 (11.8)	99 (11.0)	18 (20.9)	−0.28	0.010
Diabetes mellitus	81 (8.2)	72 (8.0)	9 (10.5)	−0.09	0.547
Chronic kidney disease	16 (1.6)	11 (1.2)	5 (5.8)	−0.25	0.005
Prior malignancy	721 (72.8)	668 (73.9)	53 (61.6)	0.26	0.021
Severity at admission					
GCS (corrected)	3.0 [3.0–6.0]	3.0 [3.0–6.0]	3.0 [3.0–3.0]	0.41	0.001
GCS category, *n* (%)				0.39	0.028
Severe (3–8)	753 (76.1)	677 (74.9)	76 (88.4)	−0.35	
Moderate (9–12)	62 (6.3)	61 (6.7)	1 (1.2)	0.29	
Mild (13–15)	85 (8.6)	81 (9.0)	4 (4.7)	0.17	
APACHE II score	24.0 [19.0–27.0]	23.0 [19.0–27.0]	28.0 [24.8–33.0]	−0.88	<0.001
ICU interventions, *n* (%)					
Mechanical ventilation	509 (51.4)	429 (47.5)	80 (93.0)	−1.15	<0.001
CRRT	15 (1.5)	11 (1.2)	4 (4.7)	−0.20	0.042
Hypertonic saline	869 (87.8)	794 (87.8)	75 (87.2)	0.02	1.000
Neurosurgical operation	758 (76.6)	695 (76.9)	63 (73.3)	0.08	0.532
Decompressive craniectomy	101 (10.2)	78 (8.6)	23 (26.7)	−0.49	<0.001
ICP/CPP monitoring					
ICP device: EVD	857 (86.6)	791 (87.5)	66 (76.7)	0.28	0.009
ICP monitoring duration, h	98.7 [39.0–156.3]	99.6 [40.6–157.1]	86.2 [18.0–148.9]	0.19	0.045
Mean ICP, mmHg	11.5 [7.5–15.8]	11.4 [7.5–15.4]	14.2 [8.6–23.1]	−0.55	<0.001
ICP variability, mmHg	4.2 [3.2–5.4]	4.2 [3.2–5.3]	5.5 [3.6–8.3]	−0.55	<0.001
ICP time > 22 mmHg, %	0.0 [0.0–6.8]	0.0 [0.0–5.4]	4.8 [0.0–50.0]	−0.65	<0.001
CPP available	950 (96.0)	873 (96.6)	77 (89.5)	0.28	0.004
Mean CPP, mmHg	78.3 [70.1–86.6]	78.5 [70.7–86.6]	72.8 [61.5–86.2]	0.46	0.004
CPP time < 60 mmHg, %	3.3 [0.0–16.7]	2.7 [0.0–15.6]	13.8 [0.0–40.0]	−0.54	<0.001
ICU course					
ICU length of stay, days	4.9 [1.8–10.7]	4.7 [1.8–10.4]	6.7 [3.4–12.9]	−0.12	0.006

Data are median [IQR] or *n* (%). SMD, standardized mean difference (survivors vs. non-survivors; |SMD| > 0.1 indicates imbalance). *p* by Mann–Whitney U (continuous) or χ^2^ (categorical). Comorbidities were derived from structured CDW coding and are likely under-ascertained (e.g., hypertension, diabetes); prior malignancy reflects this cancer center population. GCS categories do not sum to group totals owing to missing admission GCS (*n* = 90). “Variability” = within-patient SD. Abbreviations: APACHE II, Acute Physiology and Chronic Health Evaluation II; CDW, clinical data warehouse; CPP, cerebral perfusion pressure; CRRT, continuous renal replacement therapy; EVD, external ventricular drain; GCS, Glasgow Coma Scale; ICP, intracranial pressure; IQR, interquartile range; SD, standard deviation; SMD, standardized mean difference. Hypertonic saline, neurosurgical operation, and decompressive craniectomy were ascertained from institutional order and procedure records.

**Table 2:** Association of ICP/CPP summary metrics with 28-day mortality and poor neurological outcome (Glasgow Outcome Scale 1–3).

	28-Day Mortality		Poor Neurological Outcome (GOS 1–3)	
Predictor (per +1 SD)	Adjusted OR (95% CI)	*p*	Adjusted OR (95% CI)	*p*
Mean ICP	1.57 (1.26–1.97)	<0.001	1.29 (1.05–1.58)	0.017
ICP variability	1.59 (1.32–1.93)	<0.001	1.66 (1.30–2.11)	<0.001
ICP peaks (% time > 22 mmHg)	1.53 (1.26–1.85)	<0.001	1.26 (1.05–1.51)	0.014
Mean CPP	0.62 (0.48–0.80)	<0.001	0.90 (0.74–1.10)	0.29
CPP hypoperfusion (% time < 60 mmHg)	1.66 (1.33–2.06)	<0.001	1.14 (0.94–1.38)	0.20
CPP variability	1.22 (0.93–1.59)	0.152	1.25 (1.04–1.52)	0.02
Mutual adjustment (ICP vs. CPP variability, CPP cohort)				
ICP variability—mutually adj. for CPP variability	1.46 (1.20–1.78)	<0.001	1.52 (1.22–1.90)	<0.001
CPP variability—mutually adj. for ICP variability	1.10 (0.84–1.45)	0.480	1.16 (0.94–1.42)	0.16

Adjusted odds ratios are per +1 SD of each metric (logistic regression), from a complete-case sample per predictor, shown for 28-day mortality and poor neurological outcome (GOS 1–3). Adjusted models include age, sex, APACHE II, admission GCS (corrected), and diagnosis category. “Variability” = within-patient SD. The mutual adjustment rows enter ICP and CPP variability simultaneously (CPP cohort); for both outcomes, only ICP variability remains significant, indicating that the variability signal is ICP-specific. Abbreviations: APACHE II, Acute Physiology and Chronic Health Evaluation II; CI, confidence interval; CPP, cerebral perfusion pressure; GCS, Glasgow Coma Scale; GOS, Glasgow Outcome Scale; ICP, intracranial pressure; OR, odds ratio; SD, standard deviation.

**Table 3:** Incremental predictive value over a clinical base model.

Panel A. 28-Day Mortality (*n* = 856, 70 Events)					
Model	C-Statistic (95% CI)	ΔC	LR χ^2^ (df); *p*	IDI (*p*)	NRI
M0: Clinical base	0.828 (0.773–0.885)		reference		
M1: +mean ICP	0.830 (0.775–0.887)	+0.002	8.8 (1); 0.003	0.027 (0.061)	+0.18 (−0.13 to +0.59)
M2: +ICP variability	0.839 (0.790–0.897)	+0.009	11.6 (1); <0.001	0.026 (0.063)	+0.51 (+0.06 to +0.74)
M3: +ICP peaks (% >22)	0.840 (0.795–0.898)	+0.001	0.3 (1); 0.562	0.001 (0.746)	+0.19 (−0.27 to +0.46)
M4: +CPP burden (% <60)	0.842 (0.801–0.900)	+0.002	4.1 (1); 0.043	0.010 (0.164)	+0.33 (−0.04 to +0.61)
**Panel B. Poor Neurological Outcome, GOS 1–3 (*n* = 833, 194 Events)**					
**Model**	**C-Statistic (95% CI)**	**ΔC**	**LR χ^2^ (df); *p***	**IDI (*p*)**	**NRI**
M0: Clinical base	0.835 (0.807–0.873)		reference		
M1: +mean ICP	0.835 (0.808–0.874)	−0.000	2.8 (1); 0.097	0.004 (0.158)	+0.03 (−0.17 to +0.27)
M2: +ICP variability	0.844 (0.818–0.882)	+0.009	20.1 (1); <0.001	0.022 (0.009)	+0.20 (−0.04 to +0.42)
M3: +ICP peaks (% >22)	0.844 (0.818–0.882)	+0.000	0.6 (1); 0.433	0.001 (0.624)	+0.31 (−0.27 to +0.48)
M4: +CPP burden (% <60)	0.844 (0.819–0.883)	+0.000	0.3 (1); 0.571	0.000 (0.845)	+0.06 (−0.17 to +0.28)

Nested logistic models on a common complete-case sample. M0 (clinical base) = age, sex, APACHE II, admission GCS (corrected), diagnosis. Predictors added sequentially: M1, mean ICP; M2, ICP variability; M3, ICP peaks (% time >22 mmHg); M4, CPP hypoperfusion (% time <60 mmHg). C-statistic 95% CI by bootstrap (600 resamples). ΔC, change in C vs. preceding model; LR, likelihood ratio test vs. preceding model; IDI, integrated discrimination improvement (*p*, Pencina asymptotic test); NRI, continuous net reclassification improvement vs. preceding model. “Variability” = within-patient SD. Abbreviations: APACHE II, Acute Physiology and Chronic Health Evaluation II; CI, confidence interval; CPP, cerebral perfusion pressure; GCS, Glasgow Coma Scale; ICP, intracranial pressure; IDI, integrated discrimination improvement; LR, likelihood ratio; NRI, net reclassification improvement. NRI values are shown with 95% confidence intervals from 1000 bootstrap resamples. The continuous NRI is unstable when events are few, and incremental fit inference rests primarily on the likelihood ratio test.

## Data Availability

The data presented in this study are available on reasonable request from the corresponding author. The data are not publicly available due to privacy and ethical restrictions.

## Supplementary Materials

The following supporting information can be downloaded at: https://www.mdpi.com/article/10.3390/jcm15187056/s1, Figure S1: Study flow; Figure S2: Standard deviation versus coefficient of variation as the variability metric; Figure S3: First-72 h sensitivity analysis (immortal time bias); Figure S4: ICP variability and 28-day mortality by diagnosis; Table S1: Definitions of ICP/CPP metrics; Table S2: CPP by category and 28-day mortality (dose–response); Table S3: All level and variability metrics vs. 28-day mortality; Table S4: E-values for unmeasured confounding; Table S5: CPP cohort selection and inverse probability weighting; Table S6: Adjusted association of metrics with mortality outcomes; Table S7: Distribution of the Glasgow Outcome Scale (GOS) at discharge and overlap with mortality endpoints; Table S8: Associations with poor neurological outcome (GOS 2–3) restricted to patients alive at hospital discharge; Table S9: Alternative thresholds for ICP peak burden; Table S10: Sensitivity analyses adjusting for monitoring device and for treatment exposures; Table S11: Multiply imputed incremental models (MICE, m = 20); Table S12: Multiplicity correction for the twelve primary association tests. The Supplementary Methods and the completed STROBE checklist for cohort studies are also provided in the Supplementary Materials.
