# Supplementary material for: Intracranial Pressure Variability and Low Cerebral Perfusion Pressure Burden as Prognostic Markers Beyond Mean Pressure in Neurocritically Ill Patients
Source: J Clin Med. 2026 Sep 11;15(18):7056. doi: 10.3390/jcm15187056 (PMC13607955; doi:10.3390/jcm15187056)

Supplementary Materials

ICP and CPP variability and burden in neurocritical care. Multivariable models adjusted for age, sex, APACHE II, admission GCS (corrected), and diagnosis. “Variability” = within-patient standard deviation (SD).

Supplementary Methods

Model performance and incremental value. C-statistics and their 95% confidence intervals were obtained by bootstrap resampling (600 resamples), refitting each nested model in every resample. The integrated discrimination improvement (IDI) is the difference in mean predicted risk between events and non-events for the new versus the preceding model, tested with the Pencina asymptotic method. The continuous (category-free) net reclassification improvement (NRI) counts the proportions of events whose predicted risk rises and of non-events whose predicted risk falls under the new model; 95% confidence intervals for the NRI were obtained from 1,000 bootstrap resamples in this revision, because the continuous NRI is unstable when events are few. Restricted cubic splines used a natural cubic basis with three degrees of freedom and knots at quantile-based positions. Missing covariates were addressed by multiple imputation by chained equations (m=20 imputations; estimates pooled with Rubin’s rules), rare-event models used Firth’s penalized likelihood, selection into the CPP cohort was modeled by inverse-probability-of-selection weighting, and robustness to unmeasured confounding was quantified with E-values.

Multiplicity correction. For the twelve pre-specified adjusted exposure–outcome association tests of Table 2 (main text; six pressure metrics against the two outcomes, identical models and adjustment set), P values were additionally adjusted with the Holm step-down procedure, which controls the family-wise error rate, and with the Benjamini–Hochberg procedure, which controls the false discovery rate. Results are reported in Table S12. All analyses outside this family (dose–response, mutual-adjustment, incremental, and sensitivity analyses) were exploratory.

Table S1. Definitions of ICP/CPP metrics

Metric	Definition
Mean	Within-patient mean of hourly values (mmHg)
Variability (SD)	Within-patient standard deviation of hourly values (mmHg) — primary variability metric
Coefficient of variation (CV)	SD ÷ mean (dimensionless); reported for comparison only, not used as a primary metric
MAG	Mean absolute gradient — mean of |differences| between successive readings (mmHg/h)
RMSSD	Root mean square of successive differences between readings (mmHg)
ICP peaks (% time >22)	Proportion of monitored time with ICP >22 mmHg (%)
ICP dose >22	Area under the ICP curve above 22 mmHg (mmHg·h)
CPP hypoperfusion (% time <60)	Proportion of monitored time with CPP <60 mmHg (%)

All metrics were computed per patient from hourly values over the monitoring period (and, in sensitivity analyses, over the first 72 h).

Table S2. CPP by category and 28-day mortality (dose–response)

Mean CPP (mmHg)	n (deaths)	Crude OR (95% CI)	Adjusted OR (95% CI)	P*
70–80 (ref)	265 (15)	1.00 (reference)	1.00 (reference)	
<60	65 (16)	5.44 (2.52–11.73)	6.62 (2.51–17.42)	<0.001
60–70	139 (14)	1.87 (0.87–3.99)	1.85 (0.78–4.37)	0.162
80–90	235 (14)	1.06 (0.50–2.24)	1.12 (0.49–2.54)	0.790
≥90	160 (13)	1.47 (0.68–3.18)	1.37 (0.59–3.19)	0.466

**P for adjusted model vs the 70–80 mmHg reference. Excess risk is confined to hypoperfusion (<60 mmHg); high CPP (≥90) is not significantly harmful after adjustment, supporting the interpretation that low perfusion — not high CPP — drives risk.*

Table S3. All level and variability metrics vs 28-day mortality

Metric	n	Crude OR (95% CI)	P	Adjusted OR (95% CI)	P
ICP mean	900	1.67 (1.39–2.01)	<0.001	1.57 (1.26–1.97)	<0.001
ICP median	900	1.59 (1.33–1.90)	<0.001	1.51 (1.22–1.88)	<0.001
ICP SD (variability)	880	1.67 (1.36–2.05)	<0.001	1.59 (1.32–1.93)	<0.001
ICP CV	872	1.01 (0.81–1.28)	0.906	1.00 (0.76–1.32)	1.000
ICP range	900	1.28 (1.05–1.57)	0.013	1.29 (1.03–1.62)	0.024
ICP IQR	900	1.36 (1.13–1.65)	0.001	1.33 (1.11–1.59)	0.002
ICP MAG	880	1.70 (1.29–2.24)	<0.001	1.52 (1.09–2.12)	0.014
ICP RMSSD	880	1.74 (1.37–2.20)	<0.001	1.54 (1.17–2.02)	0.002
ICP % time >22	900	1.63 (1.39–1.91)	<0.001	1.53 (1.26–1.85)	<0.001
ICP dose>22	880	1.39 (1.16–1.67)	<0.001	1.32 (1.11–1.56)	0.002
CPP mean	864	0.59 (0.47–0.73)	<0.001	0.62 (0.48–0.80)	<0.001
CPP median	864	0.63 (0.50–0.78)	<0.001	0.67 (0.51–0.86)	0.002
CPP SD (variability)	837	1.20 (0.96–1.51)	0.107	1.22 (0.93–1.59)	0.152
CPP CV	837	2.47 (1.45–4.23)	<0.001	2.34 (1.29–4.26)	0.005
CPP range	864	0.94 (0.74–1.21)	0.644	0.94 (0.71–1.25)	0.689
CPP IQR	864	1.10 (0.88–1.38)	0.415	1.07 (0.82–1.40)	0.623
CPP MAG	837	1.14 (0.92–1.43)	0.237	1.04 (0.79–1.37)	0.753
CPP RMSSD	837	1.19 (0.96–1.48)	0.121	1.15 (0.88–1.51)	0.306
CPP % time <60	864	1.59 (1.33–1.90)	<0.001	1.66 (1.33–2.06)	<0.001
CPP % time <70	864	1.48 (1.18–1.85)	<0.001	1.53 (1.17–1.99)	0.002

OR per +1 SD. CV rows (shaded) illustrate why SD is preferred: ICP CV is null (mean-normalization cancels the variability signal), whereas CPP CV is spuriously inflated (it tracks low perfusion; see Figure S2). RMSSD, an alternative variability metric, reproduces the ICP variability signal.

Table S4. E-values for unmeasured confounding

Predictor	Outcome	Adjusted OR	E-value (point)	E-value (CI)
ICP variability	28-day mortality	1.59 (1.32–1.93)	2.40	1.88
ICP variability	Poor neuro outcome	1.66 (1.30–2.11)	2.22	1.73
CPP hypoperfusion (%<60)	28-day mortality	1.66 (1.33–2.06)	2.51	1.91
CPP hypoperfusion (%<60)	Poor neuro outcome	1.14 (0.94–1.38)	1.44	1.00
Mean ICP	28-day mortality	1.57 (1.26–1.97)	2.36	1.76
Mean ICP	Poor neuro outcome	1.29 (1.05–1.58)	1.70	1.23

E-value = minimum strength of association (risk-ratio scale) that an unmeasured confounder would need with both the exposure and the outcome to fully explain the observed estimate; the second column is the E-value for the confidence limit closest to the null. Larger values indicate greater robustness.

Table S5. CPP cohort selection and inverse-probability weighting

Variable	CPP available (n=950)	CPP unavailable (n=40)	SMD
Age	50.93	56.13	-0.32
APACHE II	22.60	23.35	-0.10
Admission GCS	5.12	4.28	0.27
Mean ICP	12.63	12.61	0.00
ICP variability	4.67	4.74	-0.02
ICP monitoring h	98.14	16.17	1.98
ICU LOS	7.66	3.12	0.70
28-day death	0.08	0.23	-0.40

Inverse-probability-weighted sensitivity:

CPP hypoperfusion (%<60) → 28-day mortality	Adjusted OR (95% CI) per +1 SD
Unweighted (CPP cohort)	1.60 (1.28–2.01)
IPW-weighted	1.61 (1.29–2.01)

Values are means. Patients with derivable CPP (n=950) differed modestly from those without (n=40), consistent with a monitoring-selection effect. After inverse-probability-of-availability weighting, the CPP hypoperfusion estimate was essentially unchanged, indicating selection did not materially bias the CPP association. (Weighted CI approximate.)

Table S6. Adjusted association of metrics with mortality outcomes

Predictor (per +1 SD)	28-day mortality	All-cause mortality during follow-up	ICU mortality
Mean ICP	1.57 (1.26–1.97); <0.001	1.16 (1.00–1.35); 0.056	1.66 (1.29–2.13); <0.001
ICP variability	1.59 (1.32–1.93); <0.001	1.35 (1.15–1.59); <0.001	1.58 (1.29–1.92); <0.001
ICP peaks (% >22)	1.53 (1.26–1.85); <0.001	1.08 (0.93–1.25); 0.292	1.68 (1.34–2.10); <0.001
Mean CPP	0.62 (0.48–0.80); <0.001	0.84 (0.71–0.98); 0.030	0.61 (0.45–0.82); 0.001
CPP hypoperfusion (% <60)	1.66 (1.33–2.06); <0.001	1.16 (0.99–1.35); 0.072	1.69 (1.30–2.18); <0.001
CPP variability	1.22 (0.93–1.59); 0.152	1.12 (0.96–1.31); 0.161	1.60 (1.16–2.20); 0.004

Adjusted OR per +1 SD (95% CI); P. Estimates are strongest for 28-day mortality and attenuate for in-hospital mortality, which is diluted by late (predominantly tumor) deaths (see main text). ICP variability remains significant across outcomes. The middle column, previously labeled in-hospital mortality, has been relabeled: on date-level adjudication it represents all-cause mortality at any time during the available follow-up (n=271, including deaths after hospital discharge); only 96 patients (9.7%) died before hospital discharge, and these correspond exactly to GOS 1 (Table S7).

Table S7. Distribution of the Glasgow Outcome Scale (GOS) at discharge and overlap with mortality endpoints

GOS at discharge	n	Died before hospital discharge, n	Died at any time during follow-up, n
1 (death)	96	96	96
2 (persistent vegetative state)	10	0	3
3 (severe disability)	112	0	30
4 (moderate disability)	173	0	44
5 (good recovery)	526	0	71
Not classifiable	73	0	27
Total	990	96	271

GOS 1 corresponded exactly to death before hospital discharge (96/96). Of 218 patients with poor neurological outcome (GOS 1–3), 122 were alive at hospital discharge (GOS 2–3). Deaths during follow-up after discharge occurred a median of approximately one year later in patients discharged with GOS 4–5 and were predominantly related to the underlying disease.

Table S8. Associations with poor neurological outcome (GOS 2–3) restricted to patients alive at hospital discharge

Predictor (per +1 SD)	Adjusted OR (95% CI)	P
ICP variability	1.26 (1.00–1.58)	0.050
Mean ICP	0.93 (0.71–1.22)	0.611
ICP peaks (% time >22 mmHg)	1.02 (0.78–1.33)	0.882
CPP hypoperfusion (% time <60 mmHg)	0.71 (0.50–0.99)	0.042

Patients who died before hospital discharge (n=96) were excluded; outcome = GOS 2–3 versus 4–5 (n=771 with complete covariates for ICP variability; 115 events). Adjusted for age, sex, APACHE II, admission GCS, and diagnosis. Survivor-restricted analyses condition on survival and are therefore susceptible to index-event (collider-stratification) bias; the inverse association of CPP hypoperfusion burden is consistent with such bias and should not be interpreted as protection. These analyses are reported for transparency; the pre-specified secondary outcome (GOS 1–3) remains the primary basis of inference.

Table S9. Alternative thresholds for ICP peak burden

Threshold (% monitored time above)	28-day mortality, aOR (95% CI); P	Poor neurological outcome, aOR (95% CI); P
>20 mmHg	1.55 (1.26–1.91); <0.001	1.23 (1.02–1.49); 0.031
>22 mmHg (primary)	1.53 (1.26–1.85); <0.001	1.26 (1.05–1.51); 0.014
>25 mmHg	1.43 (1.19–1.73); <0.001	1.23 (1.03–1.47); 0.022
>30 mmHg	1.45 (1.21–1.73); <0.001	1.26 (1.05–1.50); 0.011

aOR per +1 SD, adjusted for age, sex, APACHE II, admission GCS, and diagnosis. Associations were consistent across thresholds, indicating that the guideline-derived 22 mmHg cutoff did not drive the results.

Table S10. Sensitivity analyses adjusting for monitoring device and for treatment exposures

Model	Predictor	28-day mortality, aOR (95% CI); P	Poor neurological outcome, aOR (95% CI); P
+ Monitoring device	ICP variability	1.58 (1.30–1.91); <0.001	1.65 (1.30–2.09); <0.001
+ Treatment exposures	ICP variability	1.55 (1.28–1.88); <0.001	1.62 (1.24–2.10); <0.001
+ Treatment exposures	CPP hypoperfusion (% <60)	1.71 (1.35–2.16); <0.001	—
Excluding lumbar drain (n = 978)	ICP variability	1.59 (1.32–1.93); <0.001	1.66 (1.31–2.11); <0.001
Excluding lumbar drain (n = 978)	ICP peak burden (% time >22 mmHg)	1.51 (1.24–1.84); <0.001	1.26 (1.05–1.52); 0.014
Excluding lumbar drain (n = 978)	CPP hypoperfusion (% time <60 mmHg)	1.67 (1.34–2.08); <0.001	1.15 (0.94–1.39); 0.176

Base adjustment: age, sex, APACHE II, admission GCS, diagnosis. Device model additionally includes the primary ICP device (EVD 857 [86.6%]; epidural [Spiegelberg] 115 [11.6%]; lumbar drain 12 [1.2%]; intraparenchymal [Camino] 6 [0.6%]); device was not independently associated with outcome (EVD vs other: aOR 0.69, P=0.25). Treatment model additionally includes hypertonic saline (any, 87.8%), mechanical ventilation (51.4%), continuous renal replacement therapy (1.5%), any neurosurgical operation during the index admission (76.6%), and decompressive craniectomy (10.2%), ascertained from institutional order and procedure records. Lumbar-drain–excluded models omit the 12 patients (1.2%) in whom ICP was charted from a lumbar drain; one of the twelve died within 28 days.

Table S11. Multiply imputed incremental models (MICE, m=20)

Added term (sequential)	28-day mortality (N=990), pooled aOR (95% CI); P	Poor neurological outcome (N=917), pooled aOR (95% CI); P
+ Mean ICP	1.61 (1.30–1.99); <0.001	1.28 (1.06–1.56); 0.012
+ ICP variability	1.39 (1.13–1.70); 0.002	1.70 (1.30–2.22); <0.001
+ ICP peak burden	1.14 (0.78–1.65); 0.496	1.06 (0.76–1.48); 0.716
+ CPP hypoperfusion burden	1.28 (0.94–1.75); 0.115	0.88 (0.68–1.15); 0.356

Sequential models as in Table 3, refitted in multiply imputed data (chained equations, m=20; Rubin's rules). Compared with the complete-case analysis, the added contribution of ICP variability was materially unchanged for both outcomes, whereas the marginal contribution of CPP hypoperfusion burden to 28-day mortality was attenuated (complete-case likelihood-ratio P=0.043; pooled Wald P=0.115). Mean C-

statistics ranged from 0.834 (clinical base + mean ICP) to 0.843 (full model) for 28-day mortality.

Table S12. Multiplicity correction for the twelve primary association tests

Outcome	Predictor (per +1 SD)	Adjusted OR (95% CI)	P (unadjusted)	P (Holm)	P (BH-FDR)
28-day mortality	Mean ICP	1.57 (1.26–1.97)	<0.001	<0.001	<0.001
28-day mortality	ICP variability	1.59 (1.32–1.93)	<0.001	<0.001	<0.001
28-day mortality	ICP peaks (% time >22 mmHg)	1.53 (1.26–1.85)	<0.001	<0.001	<0.001
28-day mortality	Mean CPP	0.62 (0.48–0.80)	<0.001	0.002	<0.001
28-day mortality	CPP hypoperfusion (% time <60 mmHg)	1.66 (1.33–2.06)	<0.001	<0.001	<0.001
28-day mortality	CPP variability	1.22 (0.93–1.59)	0.152	0.456	0.183
Poor neurological outcome	Mean ICP	1.29 (1.05–1.58)	0.017	0.085	0.025
Poor neurological outcome	ICP variability	1.66 (1.30–2.11)	<0.001	<0.001	<0.001
Poor neurological outcome	ICP peaks (% time >22 mmHg)	1.26 (1.05–1.51)	0.014	0.084	0.024
Poor neurological outcome	Mean CPP	0.90 (0.74–1.10)	0.294	0.456	0.294
Poor neurological outcome	CPP hypoperfusion (% time <60 mmHg)	1.14 (0.94–1.38)	0.197	0.456	0.214
Poor neurological outcome	CPP variability	1.25 (1.04–1.52)	0.020	0.085	0.027

The family comprises the twelve pre-specified adjusted exposure–outcome tests of Table 2 (main text): six pressure metrics (per +1 SD; identical models and adjustment set—age, sex, APACHE II score, admission GCS, and diagnostic category) against 28-day mortality and poor neurological outcome. The Holm step-down procedure controls the family-wise error rate; the Benjamini–Hochberg procedure controls the false discovery rate. The primary hypothesis—ICP variability and 28-day mortality—remains significant even under Bonferroni correction (adjusted P<0.001). Analyses beyond this family (dose–response, mutual-adjustment, incremental, and sensitivity analyses) were exploratory.

Supplementary Figure legends

Figure S1. Study flow. Of 14,572 ICU patients screened, 992 had ICP monitoring; 990 formed the analytic cohort. Sub-analyses used the CPP-available subgroup (n=950), discharge-GOS–classifiable subgroup (n=917), and complete-covariate sample for incremental models (n=856).

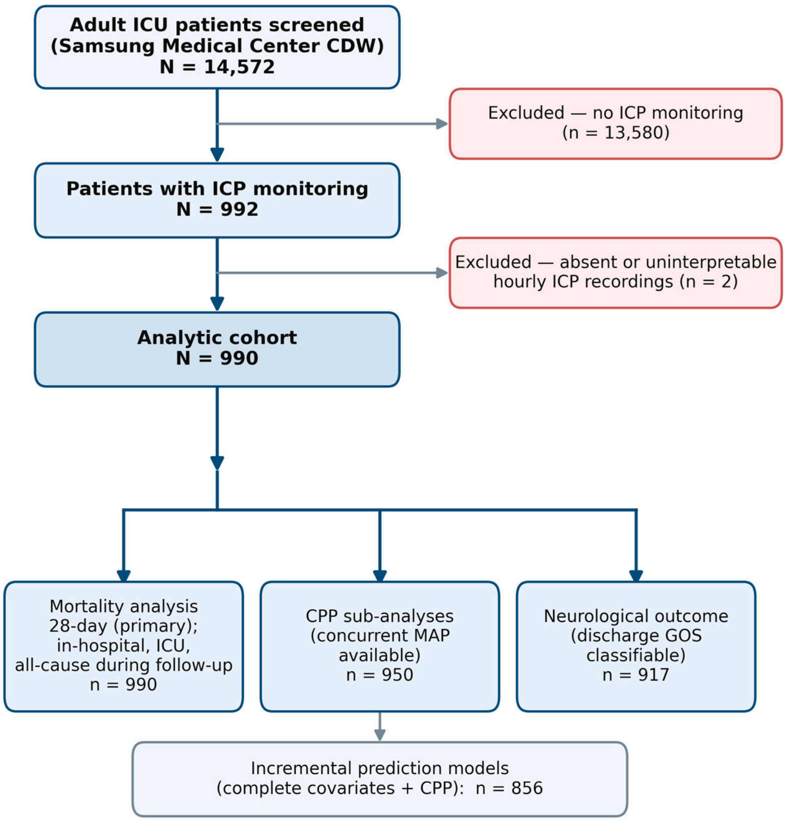
Adult ICU patients screened
(Samsung Medical Center CDW)
N = 14,572
Excluded — no ICP monitoring
(n = 13,580)
Patients with ICP monitoring
N = 992
Excluded — absent or uninterpretable
hourly ICP recordings (n = 2)
Analytic cohort
N = 990
Mortality analysis
28-day (primary);
in-hospital, ICU,
all-cause during follow-up
n = 990
CPP sub-analyses
(concurrent MAP
available)
n = 950
Neurological outcome
(discharge GOS
classifiable)
n = 917
Incremental prediction models
(complete covariates + CPP): n = 856

Figure S2. Standard deviation versus coefficient of variation as the variability metric. (a) Adjusted OR per +1 SD: ICP variability (SD) predicts both outcomes, but ICP CV is null and CPP CV is spuriously inflated. (b) Patients in the highest CPP-CV tertile have the lowest mean CPP and the greatest hypoperfusion burden, showing that CPP CV captures low perfusion rather than variability.

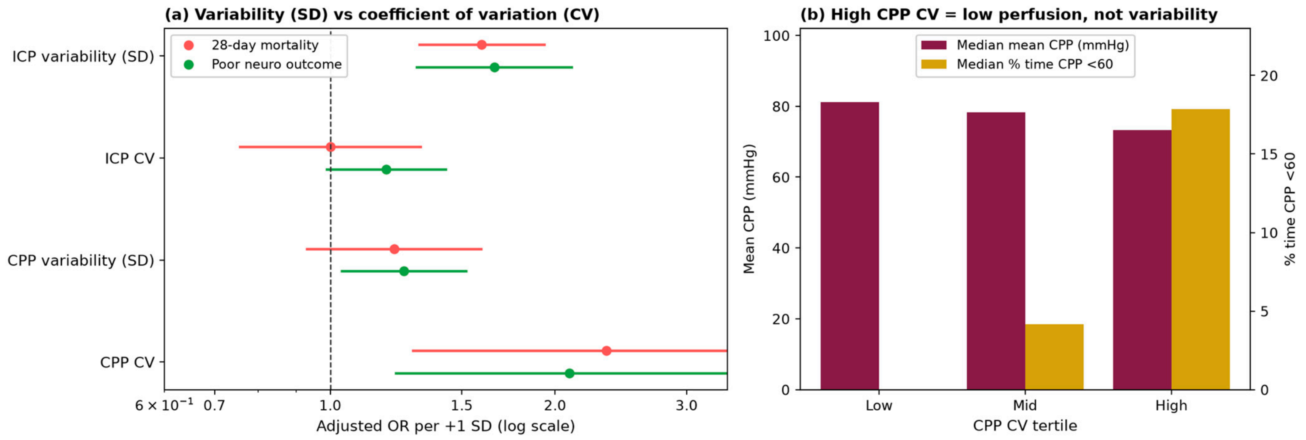

Figure S3. First-72 h sensitivity analysis (immortal-time bias). Adjusted OR per +1 SD for 28-day mortality using whole-stay versus first-72 h exposure windows are closely concordant, indicating the associations are not driven by longer monitoring in survivors.

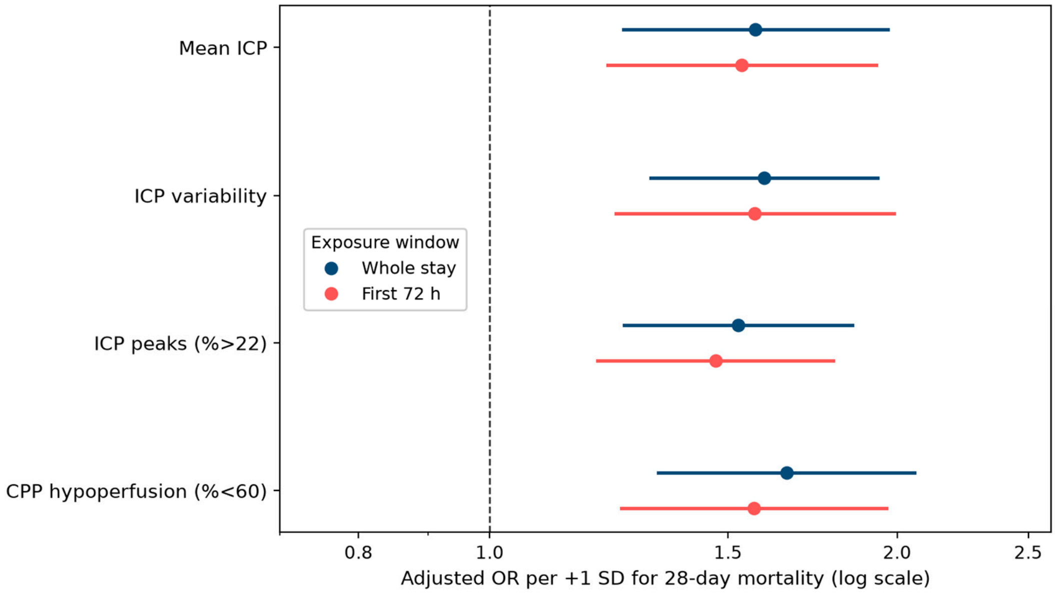

Figure S4. ICP variability and 28-day mortality by diagnosis. The adjusted association is consistent in brain-tumor and non-tumor patients (interaction P = 0.97), indicating the variability signal is not diagnosis-specific.

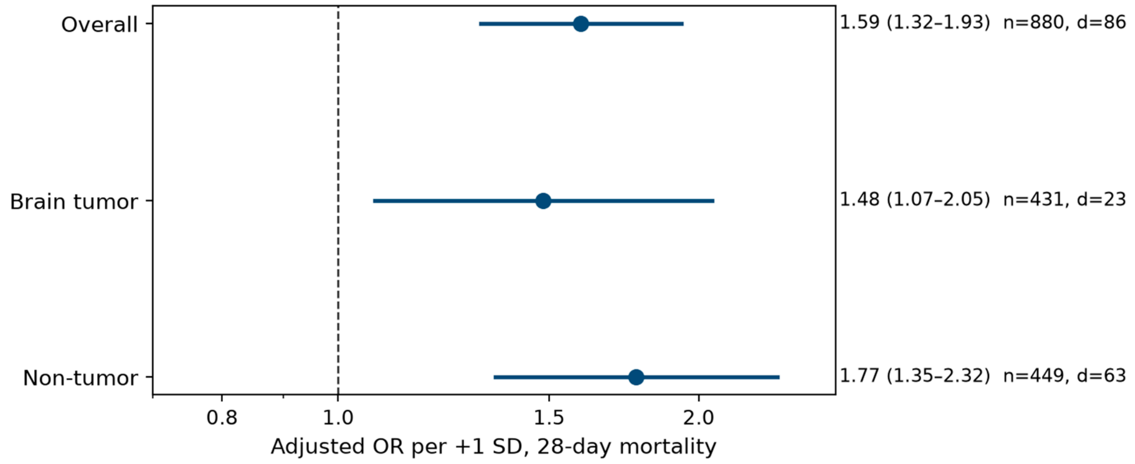

Supplement: Supplementary file 1 [file jcm-15-07056-s001.zip › jcm-4489495-supplementary.pdf]
